# An Analysis of the Displacements in 3D-Printed PLA Acoustic Guitars

**DOI:** 10.3390/polym16152108

**Published:** 2024-07-24

**Authors:** Álvaro Burgos-Pintos, Francisco Fernández-Zacarías, Pedro F. Mayuet, Ricardo Hernández-Molina, Lucía Rodríguez-Parada

**Affiliations:** 1 Department of Mechanical Engineering & Industrial Design, Faculty of Engineering, University of Cadiz, Av. University of Cadiz 10, 11519 Puerto Real, Spain; alvaro.burgospintos@alum.uca.es (Á.B.-P.); francis-co.fernandez@uca.es (F.F.-Z.); ricardo.hernandez@uca.es (R.H.-M.); lucia.rodriguez@uca.es (L.R.-P.); 2 Acoustic Engineering Laboratory, University of Cadiz, 11519 Cadiz, Spain

**Keywords:** additive manufacturing, acoustic guitar, product design, vibration testing, 3D printing, PLA, methodology, parameterization, simulation, musical instrument

## Abstract

This study focuses on the analysis of the displacements generated in 3D-printed acoustic guitar tops. Specifically, the influence of 3D printing direction parameters on the vibrational behavior of a guitar top designed for polylactic acid (PLA) by analyzing five points of the top surface at a reduced scale. For this purpose, finite element tests and laboratory experiments have been carried out to support the study. After analyzing the results, it can be affirmed that the vibrational response in reduced-scale top plates can be modified and controlled by varying the printing direction angle in additive manufacturing, providing relevant information about the displacement in the vibrational response of PLA acoustic guitars. Furthermore, this work shows that the behavior of a specific acoustic guitar design can be characterized according to a specific need.

## 1. Introduction

### 1.1. The Top Plate of the Acoustic Guitar

The acoustic guitar is a chordophone instrument composed of six strings which, when plucked, begin to vibrate. This vibration is transmitted to the bridge, placed on the soundboard and main component for the generation of the sound of the instrument [1]. This cover must be structurally reinforced in order to support the tension generated by the strings, and, at the same time, correctly transmit the vibration of the strings over the entire surface of the cover, thus generating the final sound of the instrument [2,3].

**Figure 1:**
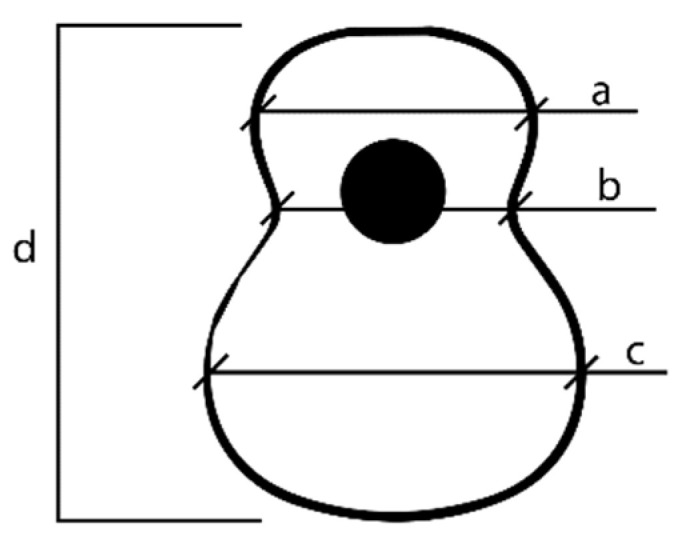
Parts of the top plate.

For its study, it is necessary to identify three main zones shown in Figure 1 that make up the soundboard of an acoustic guitar. Thus, zone “a” is the minor lobe located in the upper area of the top above the sound hole. This is followed by zone “b”, known as the waist lobe, a critical area of the design due to the concentration of stresses caused by the reduction in surface area in this area. Finally, the major lobe or main area is “c”, responsible for the generation of the sound of the instrument, since the bridge of the guitar is located there and most of the vibration of the top is generated [4]. The letter “d” is the total height of the Top Plate.

It should be noted that the design of the reinforcement associated with the top has varied throughout history depending on the knowledge of the luthier who built the instrument, although it is worth mentioning many authors such as Antonio de Torres, René Lacote, Mirecourt, or José Ramírez III, whose reinforcements—called varetajes—provided a good sound for the instrument [1,5]. In fact, their designs are still being used today in the manufacture of new acoustic guitars. In Section 2 of this work, a layout of the wiring harness in the design of the covers is shown. This arrangement is applied to the small-scale specimens vibrationally tested in this study to measure their displacements.

### 1.2. Additive Manufacturing and Its Use in the Music Industry

Nowadays, the use of industrial design for product customization is becoming increasingly important, especially through the use of new technologies such as additive manufacturing. In the music sector, this technology is facilitating the creation of new musical instruments and complex geometries that provide ergonomics and new acoustics [6,7,8]. A clear example is the family of string instruments created by MONAD, who create violins and cellos through parametric design following an organic line and away from the traditional, generating new sounds and high customization possibilities [9,10,11].

Additive manufacturing is a technology that is commonly used in the creation of new designs manufactured by fused filament fabrication (FFF) in order to generate instruments with a good acoustic response. An example of this is the trumpet designed by Marano et al., who combine different geometries by parameterization to achieve new sounds [12,13,14].

For the vibratory study of stringed instruments, there are several modal analysis studies in which the vibratory patterns can be visualized [3,15]. For this, it is necessary to excite them by means of a speaker or tapping. Thus, depending on the modal tests, they can be by continuous, impulsive, or random excitation, requiring excitation devices such as those mentioned above [16,17]. For their visualization, there are different non-intrusive techniques that do not damage the specimens and are very useful for carrying out different studies, such as the use of holographic interferometry, velocimetry, and laser Doppler vibrometry [18,19,20].

In addition, simulation tools are being used in combination with experimental tests in order to provide more value and reliability to the results. In particular, the use of finite element method (FEM) simulation for pre-calculations of the behavior of a design is essential. This aspect is very important in the work developed in this article, since studies are being carried out on the acoustic behavior of guitars using this technology, in order to observe the vibratory repercussion depending on the design of the guitars.

In the work carried out by Torres et al., shown in the work performed by E. Kaselouris et al. [21], the displacements of different harmonic covers of Stradivarius violins are studied. Thus, FEM techniques are used to analyze the displacements of these caps in their different vibratory modes, and the results obtained are reliable according to the existing literature with respect to the experimental techniques applied. They also simulate the top without the body and neck components, an important aspect to take into account when working with acoustic guitar harmonic tops in this work [22,23,24].

The work of Beattie et al. [25] concludes that print orientation influences the modulus of elasticity and flexural strength. In addition, Kumar et al. [26] concluded that there is a direct relationship between the modulus of elasticity and the natural frequency of the specimens tested. On the other hand, Lesage et al. [17] showed that the sensitivity to vibrational modes was related to planar, vertical, or rotational printing orientation. Yao et al. [27] found that the maximum tensile strength decreased as the printing angle became smaller or the layer became thicker.

The background information shows that the results of the harmonic top are influenced by multiple variables, such as the bending, type of material, thickness of the top, and direction of the wood grain, among others. Therefore, based on the background, the direction of PLA printing could be a fundamental design factor to be taken into account in the vibrational results of the harmonic top by additive manufacturing. Consequently, the objective of this work focuses on determining the influence of the printing direction on the vibrational response of the harmonic cap. Furthermore, considering that wooden soundboards are manufactured with the grain direction perpendicular to the bridge of the guitar, this should be the most suitable direction of printing direction for FFF printed soundboards.

## 2. Materials and Methods

### 2.1. Materials

The material used to print the specimens composing the sample was PLA (polylactic acid) filament of diameter 1.75 mm, Esun brand. The mechanical properties of the material were obtained from the supplier in accordance with the REACH Regulation, RoHS Certification Services, and EN 13432-2000, according to [28,29,30].

The sample used in the test to analyze the vibration behavior of the harmonic caps is composed of 10 units, at a scale of 0.6:1. Each of the caps, manufactured by FFF, corresponds to 5 different printing directions, 0°, 90°, +45°, −45°, and ±45° (see Table 1), keeping the filling and velocity parameters constant at 100% and 60 mm/s, respectively. The nozzle used was 0.4 mm in diameter, and the layer height is 0.2 mm according to Fadhil et al. [31]. Two units of each printing direction were fabricated to analyze the impact of reproducibility when fabricating by FFF, and the mean was used as the value for analysis.

Solidworks software (Solidworks 2019 SP5.1) was used to design the specimen and its subsequent 3D printing was performed with an Creality Ender 3 Pro (Shenzhen, China). This software was also used to model the vibration study by means of finite element simulation (FEM).

The specimens were harmonically excited through a loudspeaker, model ALT/IN35, whose driver is the MKS 78F-076G (3 Omh—5 W, 2.5”). The loudspeaker was connected to the different specimens, using a plastic tube attached from the loudspeaker to the surface of the top plate. For top plate vibration data collection, a Wintact WT63B handheld vibrometer was used, which covers measurement ranges from 0.1 to 1.999 mm and has the measurement ranges and accuracy detailed in Table 2.

The excitation signal was generated using NHC Tone Generator software version 3.56 and transmitted to the loudspeaker through a Steinberg UR44 sound card connected to the audio output of the PC. The recorded experimental results were imported with the MatLab R2022b program.

### 2.2. Experimental Procedure

A methodology is proposed to evaluate the impact of the printing direction on the vibrational response of the different covers. The results obtained in this study were compared with the displacements obtained by means of the finite element in-test.

**Figure 2:**
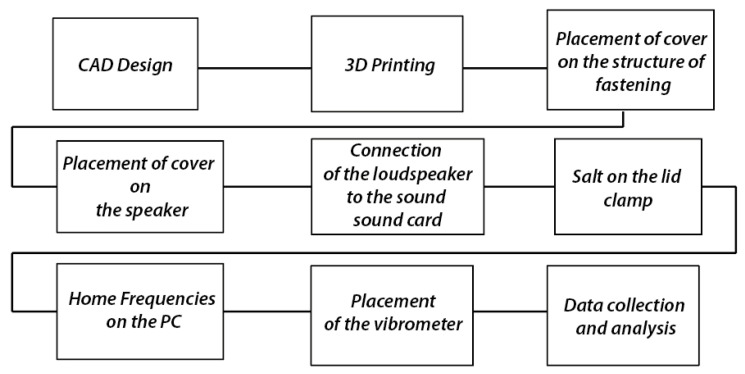
Procedure used.

The work was carried out following the steps defined in Figure 2. The reference design of the harmonic top used to create the specimens corresponds to the model created by the luthier Antonio de Torres.

**Figure 3:**
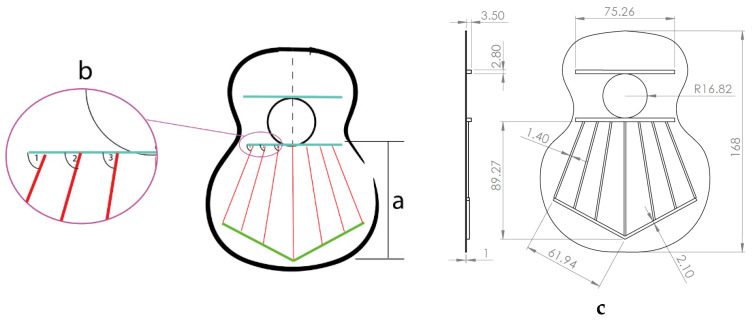
Design of guitar bracing: (**a**) harmonic rods; (**b**) fan rods fan distribution angles; (**c**) dimensions.

Figure 3 shows the bracing of the specimens, which presents a symmetrical structure, composed of a total of seven rods in the fan, with closure of the same by two rods (green lines), and two harmonic rods placed horizontally (blue lines). It should be noted that the lower lobe and waist have a horizontal rod that separates both lobes, and in the larger lobe, a series of rods in symmetrical arrangement that create what is called fan. The traditional arrangement of the fan is defined by angles 1, 2, and 3, taking as reference the horizontal axis, which corresponds to inclinations of 69°, 75°, and 84°, respectively, according to [1]. The overall size of the lid is 16.8 × 13 cm. It should be added that these caps were not subjected to any subsequent surface treatment.

**Figure 4:**
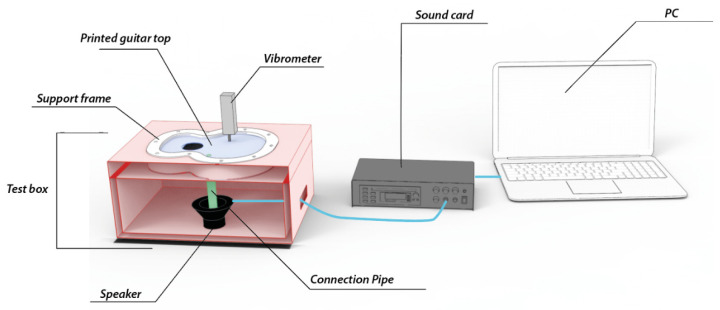
Test layout and components.

The test specimens were individually placed on a structure developed for holding the tops (Figure 4), which was made of medium-density wood strips (MDF) selected for its high thickness, thus avoiding vibrations when creating the frequency sweep to the test specimens. Frame was printed in PLA to hold the different specimens, a where they were held laterally, simulating the union with the bows of the guitars and avoiding lateral movement of the same. Finally, inside the cavity, the speaker was introduced with a Velcro connection to the base of the box, with the correct height so that the speaker’s connection tube with the top plate was the correct one to make it vibrate correctly.

The airtightness of the box where the different lids are held is not relevant in this work, since we are not looking for an acoustic analysis of the box, but only the vibration analysis of the printed harmonic lids subjected to the test.

Specifically, this study focused on the analysis of the deformations caused by the first 5 vibration modes of the different printed specimens starting at 100 Hz, to cover the lowest frequency of the guitatele. To visualize the different vibrational patterns, fine salt was sprinkled on the different caps and a frequency sweep was applied from 80 to 10,000 Hz. In this way, the frequencies associated with the first 5 vibratory modes were obtained, and displacement data were taken. The salt was spread by means of a sieve to avoid hand contact.

The location of the points on the soundboard provides relevant information about the vibration transmission over the entire surface of the soundboard. It is therefore important to choose the right points, an aspect to take into account when learning the vibration of the instrument.

**Figure 5:**
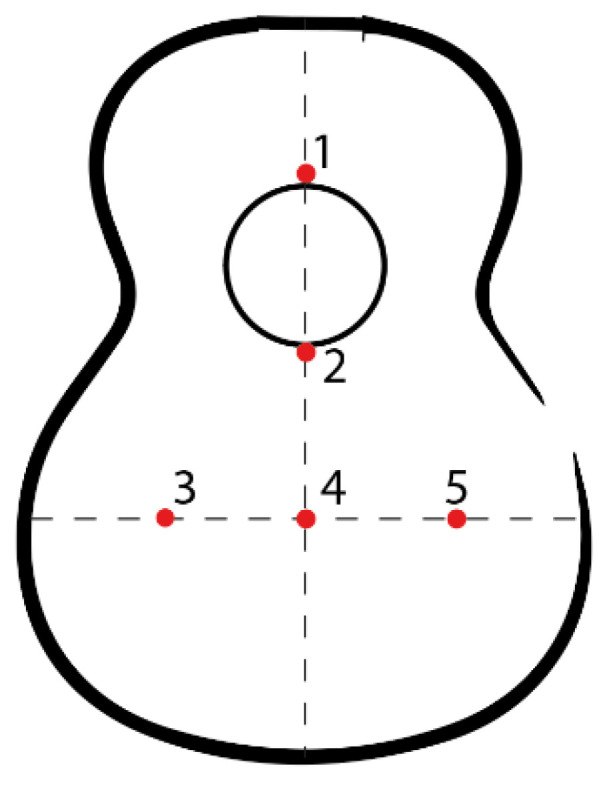
Points assessed in the linear harmonic dynamic test.

To analyze the displacements associated with each visualized vibratory pattern, 5 points of the top plate (see Figure 5) with high importance in the vibratory analysis of the same—due to the vibratory patterns visualized in the different studies carried out [5,32,33]—in which data collection points were placed to cover the surface where the vibratory patterns were created. In this work, these points were reduced, delimiting the vibratory area of the top plate.

As shown in Figure 5, points 3, 4, and 5 cover the bridge area of the guitar. Point 3 is on the left side of the top, where the bass sounds are generated, and point 5 is on the right side of the top, where the high frequencies are generated.

Displacement data were expressed as means. The relationship between the distributions of the different printing directions for each of the modes was verified using an ANOVA test. For this, a 95% confidence interval was considered (significance 5%).

### 2.3. Analysis of Vibration Result: Test Simulation by FEM

**Figure 6:**
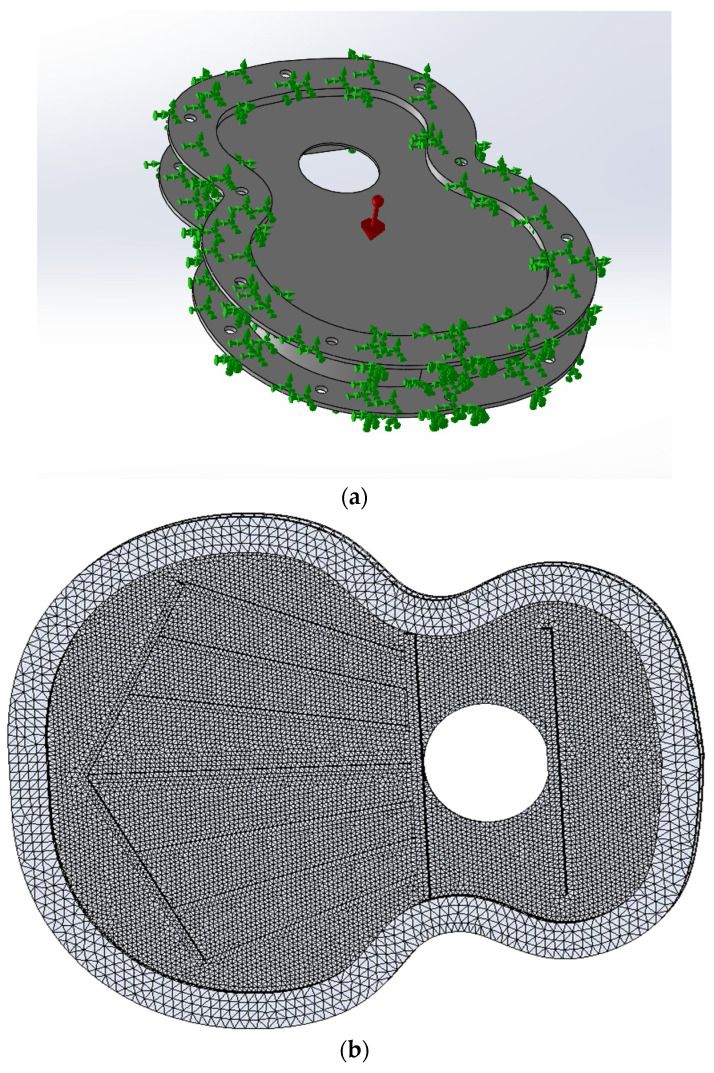
(**a**) Boundary conditions; (**b**) points evaluated in the harmonic linear dynamic test.

The purpose of performing a finite element simulation (FEM) of the vibration analysis of the harmonic cap design was to analyze the displacements of the 5 points, as shown in Figure 5. The simulation was carried out using SolidWorks software (Dassault Systems, Solidworks 2019 SP5.1) with its Simulation Premium package. First, a vibration study was created where the material used to print the caps was replicated, according to [34]. Then, a sketch was created with the position of the measurement points, and at the time of extracting the displacement data, the nodes of the mesh closest to the sketch were chosen, greatly approximating the data acquisition, Figure 6. The boundary conditions are shown in Figure 6a, where the edges holding the tested lids were replicated, as shown in Figure 4. The clamping frame has a fixed element condition, as tested experimentally. The top plate has edges attached to the clamping frames along with gravity perpendicular to the harmonic cap itself, as shown in Figure 4. The major differences in the simulated test with respect to the experimental test is the inability to replicate the printing directions.

On the other hand, it should be noted that the internal structure of the manufacturing of the specimens was not taken into account in the simulation since the objective of this work is to analyze the impact of changes in the variables associated with the manufacturing direction, in order to see the vibrational change in these variables [35,36,37,38,39,40].

### 2.4. Harmonic Cap as a Mechanical System Subjected to Vibration

The FEM system is represented by multiple differential equations, which in matrix form can be expressed as follows:

(1) $$\left[M\right]\vec{\ddot{x}}+\left[C\right]\vec{\dot{x}}+\left[k\right]\vec{x}=\vec{F}$$

where the matrices M, C, and k are square matrices of mass, damping, and stiffness, respectively, of m x, and the acceleration, velocity, displacement, and force components are vectors of the m dimension.

As a first approximation, we can consider only the existence of viscous damping, in which case, the energy dissipated by the damping can be expressed by the Raley dissipation function “R”, defined as follows:

(2) $$R=\frac{1}{2}{\vec{\dot{x}}}^{T}\left[C\right]\vec{\dot{x}}$$

The solution to the differential Equation (1) can be expressed as follows [41]:

(3) $$\vec{x}={\vec{A}}_{m}e^{\lambda_{m}t}$$

This result represents an eigenvalue and eigenvector problem. Each of the solutions $\lambda_{m}$ will be complex conjugates, and an eigenvector An corresponds to each of them.

The complex conjugate solution is usually expressed as follows:

(4) $$\lambda_{m}=a_{m}\pm j\omega_{m}$$

In FEM systems, damping is usually dispensed with, so that the solution of our system will only have an imaginary component, and it is possible to decouple the system and determine a modal mass and modal stiffness associated with each mode of vibration, so we have the following:

(5) $$\omega_{nm}^{2}=\frac{k_{m}}{m_{m}}=\frac{{\vec{A}}_{m}^{T}\left[K\right]{\vec{A}}_{m}}{{\vec{A}}_{m}^{T}\left[M\right]{\vec{A}}_{m}}$$

Each of these solutions would represent the displacements as a function of time of each of the masses when it is vibrating with its natural frequency, which is substituted by (3).

(6) $${\vec{x}}_{h}={\vec{A}}_{m}e^{\pm j\omega_{nm}t}$$

The response to an external excitation is represented by the ratio between the external excitation and the mechanical impedance:

(7) $${\vec{x}}_{p}={\left[Z\left(j\omega \right)\right]}^{-1}\vec{F}$$

where $Z\left(j\omega \right)$, neglecting the damping term, is determined as follows:

(8) $$Z\left(j\omega \right)=\left[\begin{array}{ccc} Z_{11} & \cdots & Z_{1m}\\ \vdots & \ddots & \vdots \\ Z_{m1} & \cdots & Z_{mm} \end{array}\right]=\left[\begin{array}{ccc} -\omega^{2}m_{11}+k_{11} & \cdots & -\omega^{2}m_{1m}+k_{1m}\\ \vdots & \ddots & \vdots \\ -\omega^{2}m_{m1}+k_{m1} & \cdots & -\omega^{2}m_{mm}+k_{mm} \end{array}\right]$$

The determination of the solutions will depend on the initial conditions.

## 3. Results

Two samples were printed from each direction, and after testing, it was found that the standard deviation relative to the displacement term was better than 0.02 mm. Subsequently, due to variations in the natural frequencies, influenced by the different printing directions, the frequency ranges associated with the first five vibration modes were defined: Mode 1 {110–125} Hz, Mode 2 {280–320} Hz, Mode 3 {340–365} Hz, Mode 4 {400–490} Hz, and Mode 5 {730–840} Hz.

**Figure 7:**
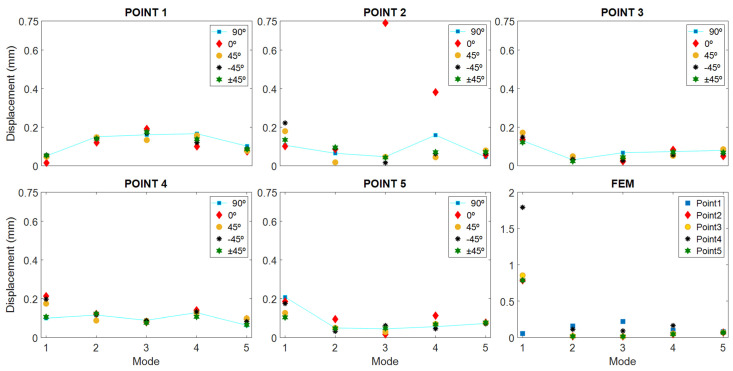
Displacement results as a function of vibration modes and print directions.

Figure 7 shows the results of displacements obtained as a function of printing direction, distributed by test points of the specimen. The simulation data have also been plotted by finite element in the lower right part of the graph, labeled “FEM”.

**Figure 8:**
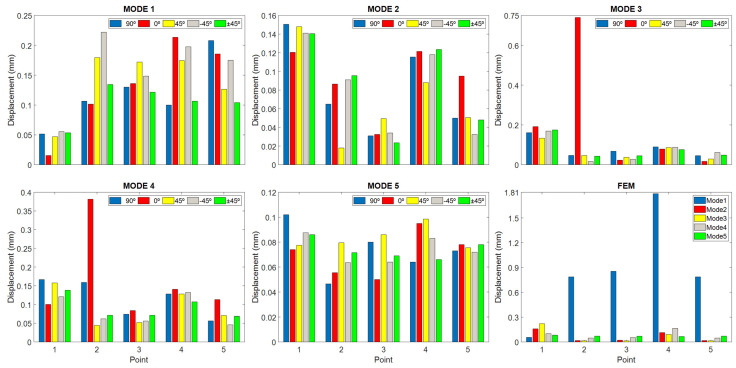
Displacement results as a function of measuring points and print directions.

Figure 8 shows the same information, but distributed by vibration modes, and a change in scale has been introduced in the ordinate axis to observe the displacement changes in greater detail. Likewise, the last “FEM” graph shows the data obtained by finite element analysis but evaluated at each test point.

In both graphs, it can be seen that the displacement deviations at each point and mode seem at first sight very contained, below 0.053 mm (see Table 3), except in the specimen printed at 0°, where point 2 in mode 3 and 4 clearly stand out from the rest. However, in this specimen, at point 1, in almost all cases, the smallest values with respect to the rest of the sample were also recorded.

Point 2 of the specimens printed at 0° is the most striking, because it is far away from the rest of the values of the sample; however, if you pay attention, there are other points of the sample where the specimen printed at 0° stands out from the rest of the specimens because of the opposite situation, for example: point 1 in mode 1, point 1 in mode 4, point 3 in mode 5, etc. Therefore, it could be thought that if the specimen printed at 0° is removed from the sample, the remaining set would show a behavior that may be quasi-independent of the printing direction. But to support this assertion, we have to turn to statistics and evaluate whether these differences are significant or not. But to support this assertion, one has to turn to statistics and evaluate whether these differences are significant or not.

According to Figure 8, the lowest values of displacement for all the specimens are in modes 2 and 5. It should be kept in mind that in vibrations, a difference of 0.1 mm is a relatively high value, and that as the frequency increases, the magnitude of the vibration decreases by the same amount of energy.

As mentioned above, looking at Figure 7 may lead to erroneous conclusions, so a statistical test was performed to compare the behavior of the displacement as a function of the printing directions in each of the modes defined in this work. The hypothesis statistical is defined as follows:

The distribution of displacement is the same across categories of mode (i.e., varying print directions).

**Figure 9:**
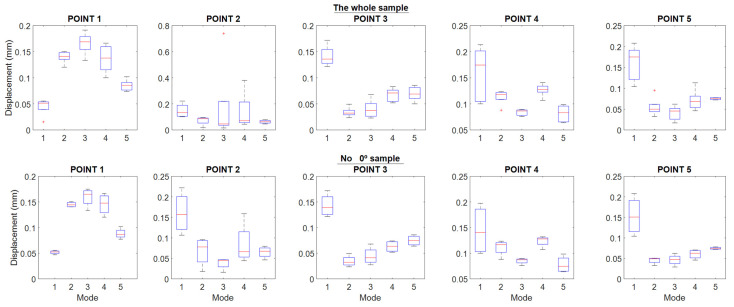
Showing the variation in point and mode of top plate direction behavior. The red line corresponds to Q2 (Median).

The results of this test are shown in Figure 9 and Table 4 and Table 5. Table 4 represents the results of all the directions studied in this work {90°, 0°, 45°, −45°, and ±45°}. As can be seen, the difference in the deviations between one printing direction and another is very significant; the exception is in point 2, where the test fails, and we cannot assure anything without enlarging the number of samples. These results can be checked in Figure 9; the five upper graphs correspond to the results of Table 4.

Examining the figure, upper part, it can be observed that at point 1 mode 1, point 2, mode 3, point 4 mode 2, and point 5 mode 2, almost all points present outlier data. Obviously, if we go back to graph 8, we can intuit that the outliers come from the specimen printed at 0°. Consequently, it remains to remove the specimen printed at 0° from the sample and recheck the result.

Figure 9, lower part, shows the indicated results once the specimen printed at 0° has been removed. As can be seen, the outliers have disappeared; however, the trends appear to have been affected very subtly. As a consequence of removing this specimen, the extremes have been affected in many of the sample displacement values.

If we look at these results in Table 5, in the “Prob > F” field, we can deduce that in this case, the results between different printing directions are very significant. With this, it should be understood that the printing directions generate trends and behaviors in the displacement depending on the very significant modes between them. Therefore, it is convenient to analyze which printing direction shades each of the modes in the most regular way; in this way, one could have the direction that makes the results less dependent on the modes.

In a following phase, it would be convenient to determine which of these directions is the one that produces the best sound quality in the instrument.

Looking at Figure 9, point 1 is the best average result achieved throughout all the modes. At this point, the best performing cover, according to Figure 7, is recorded in the 90° print, followed by the one printed with a +45° direction.

**Figure 10:**
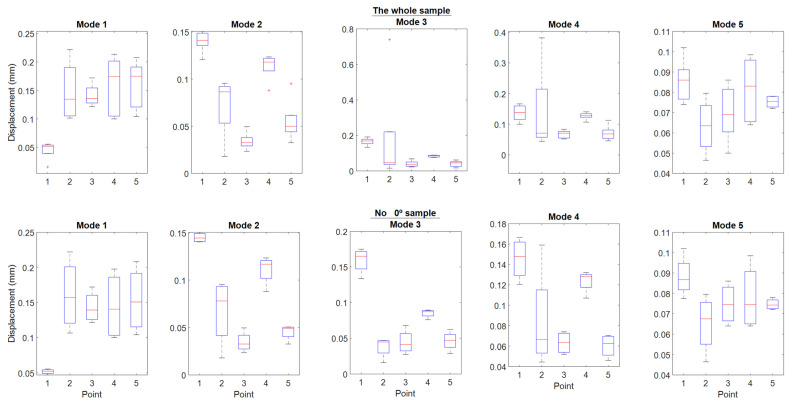
Box plot with mode and point variation of the lid direction behavior. The red line corresponds to Q2 (Median).

Figure 10 shows the statistical values separated by modes using box plots. The information that can be extracted from this figure is that modes 1 and 5 are the ones with the most uniform averages.

In general, the trend of the displacements obtained in the specimens is very similar to that of the FEM simulation.

### Comparative Analysis of FEM Test/Experimental Test Displacements

In order to comparatively analyze the results obtained in the physical tests and the simulation, the relative error was calculated. For this purpose, Equation (9) was used, according to [42], where *Er* is the relative error. In this way, the impact of the printing direction was observed.

(9) $$Er=\frac{Test\;value\;-\;simulation\;value}{test\;value}\times 100,$$

In this case, the FEM and experimental modes that more or less coincide in frequency were analyzed, i.e., mode 2 of the FEM simulation with mode 4 of the experimental analysis and mode 5 of the FEM simulation with mode 5 of the experimental analysis.

The results of the relative error are shown in Table 6. Table 6 shows the relative errors of point 4, located in the center of the major lobe above the top plate. In general, the errors are below 30%, presenting values to be taken into account for the use of the FEM in this type of test, but there are some vibratory modes and directions where this error increases.

## 4. Discussion

### 4.1. Laboratory Physical Tests

It should be noted that the low-frequency mode is conditioned by the scalability of the fabricated harmonic cap. However, this should not be a problem, insofar as it is a matter of comparing the results obtained with different printing directions, and, therefore, it is independent of the frequency limits reached, as long as similar frequency values are compared.

It should be made clear that, in the present work, the aim is not to check the quality of the tested cap, but to know if the printing direction has an influence on the vibratory displacements in the harmonic cap, in order to know if this parameter is a determining factor to be taken into account in the design of the instrument.

Figure 7, point 2 shows the displacement of the point below the mouth of the harmonic flap. At this point, the top with the +45° direction provides the smallest average displacement compared to the others, so this direction, it does not favor a large displacement. Therefore, a reinforcement in manufacturing structure perpendicular to the direction of the guitar strings in frequency ranges from 280 to 320 Hz and from 400 to 490 Hz provides high displacements compared to the other directions in the lower area of the soundboard.

At point 3, located in the left area of the larger lobe in the bridge area, the top with the best average result throughout all modes is the one printed with +45° direction, reaching the highest displacement in mode 1, with respect to the lowest one recorded with printing direction ±45°, showing that a higher internal reinforcement reduces the displacement of this one. The influence of the direction towards the low string zone and the improvement in the vibration in this zone at the low frequency of the first vibratory mode is shown. On the other hand, as we move up frequencies to “Mids” and “High-Mids”, mode 3 and 4, the fabrications with 90° and 0° present greater displacements, respectively, at point 3.

In Figure 7, “Point 4” shows the displacement at the center point of the larger lobe; except in mode 1, the behavior of the previous point is repeated where the least displacement is in the direction ±45° as the frequency increases—since it tends to move the top plate in the bass area—and creates excessive reinforcement in the larger lobe that disables the good displacement of the central area. At this point, in mode 1, with a printing direction of 0°, a displacement of slightly more than 0.2 mm is achieved, which causes the vibrations to concentrate in the central zone of the larger lobe, causing the displacement to increase, while with a direction of 90°, it achieves the smallest value. In the {400–490} Hz zone, mode 4, the largest displacement is also reached with 0°, and when increasing the frequencies to the {730–840} Hz zone, mode 5, the direction of 90° reaches the lowest value, showing again the impact of vibration in the zone of high frequencies and its relation to the direction of impression.

At point 5 in Figure 7, located in the treble area of the top, the best result in bass is presented by the 90° direction, since it acts like conventional wooden tops, which are built in the direction of the wood grain, enhancing the bass sound. On the other hand, at frequencies from 280 to 320 Hz, a 0° direction of impression favors the displacement, but on a smaller scale compared to the previous mode. As frequencies increase in the following vibratory modes, the most relevant direction of manufacture is that of −45°, observing that this direction favors the displacement and the tendency of the vibration to high frequencies, contrary to what happened with point 3, located in the opposite area of the top plate, where low frequencies are found.

Therefore, the influence of the manufacturing direction changes the displacement values of the harmonic caps tested and, therefore, their vibration behavior.

In a general analysis of the results, the 90° directions provide greater displacements in almost all the modes of point 1, located in the minor lobe; this occurs because the vibration transmission is favored by the printing lines in that direction, simulating in a certain way the behavior that is generated with wood caps, whose grain is in that direction.

Mode 1 concentrates the highest average vibrational energy of the last three strings, where the frequencies are lower (Figure 10). The points with the largest displacement are 2, 3, and 4.

In general, the −45° direction provides good results. In this sense, it is important to point out the relevance of this direction for low frequencies since it is oriented to the bass area.

Except for the atypical situation of the specimen printed at 0°, the rest of the specimens of the samples behave in a coherent way, due to the fact that the highest values of displacement occur at low frequencies (Figure 10).

### 4.2. Finite Element Testing

In the FEM simulation, it was not possible to match all the vibrational modes originated by the laboratory test for all specimens. This is because the software is not able to model the printing direction conditions. However, it has been possible to compare for those modes that do fall within the range defined in the laboratory tests.

In general, the trend of the displacements of most of the points is very close to the simulation, although in others, the errors are very high. Specifically in mode 5, compared to the printing directions of −45° and ±45°, the errors are below 12%.

In the first mode that could be compared at 230 Hz, most of the printed covers outperform the simulation by a large margin. However, in general, the trend is similar to the experimental tests, but without taking into account the internal structure of the material when varying the printing direction.

The increase in frequencies, reaching 700 Hz, causes the displacements in the top plate to be reduced, equalizing the values for the entire surface of the top plate, whose points with the greatest displacements are point 1 and 5. This is an important aspect since the area of the high strings is located in the left area of the top plate, where point 5 is, and whose behavior was observed in the experimental results.

### 4.3. Overall Assessment and Future Lines

After the analysis carried out in this work, it is necessary to point out important and relevant aspects in relation to the use of PLA and FFF in the manufacture of musical instruments.

The use of FFF manufacturing provides an important aspect in the design of acoustic guitars, due to the ability to experiment with the printing direction parameter when generating vibrations in the soundboard. A clear example is the work carried out by T. Zvoníček et al. [43], where they show the acoustic capability in the musical domain. The work addresses the acoustic properties of different materials using FFF technology. Tests are performed by varying both, nozzle diameters, and internal configurations. As a conclusion, it shows a great acoustic response of PLA compared to other materials, thus showing the capability of the material for future musical instrument designs.

Following the research field of PLA in acoustic aspects, the work carried out by S. Matei et al. [44] should be highlighted, as it shows the capacity of additive manufacturing with the use of PLA for acoustic insulation. In this work, they show the repercussion of the change in printing direction on the acoustic behavior of the different parts printed in PLA. This work is a great example of the capacity of the material and the FFF technology to generate designs with good acoustic performance.

Taking into account the works mentioned above and the results obtained, it is necessary to go deeper into the acoustic aspect of the pieces tested. Since this work focuses on a vibratory study of the top plate, and not so much on the final acoustic result. This requires future studies focused on the acoustic response of the printed tops, where the different printing parameters are tested in addition to an increase in the scale of the tops. The increase in scale in the manufacture will provide us with the ability to compare with wooden guitars on the market, making it possible to clarify the acoustic behavior of the material in a more direct way.

A disadvantage of using PLA for this purpose is its mechanical properties, which is why it is considered important to study the mechanical capacity for the design of acoustic guitars in greater depth in future work, including the development and testing of reinforced materials to increase these properties. Bearing in mind the above, it is important to emphasize that the main purpose of the current and future studies is to be able to design an acoustic guitar in a personalized way, according to the guitarist’s taste.

## 5. Conclusions

Based on the results obtained, the following can be stated:

- The printing directions by additive manufacturing indicates that it allows for modifying vibrational behavior of the harmonic cap.
- Modifying the vibratory behavior adds a new possibility for the design of acoustic guitars, using FFF printing as a fundamental tool to customize the instrument according to the guitarist’s tastes, since it will be possible to parameterize and use the printing parameters for a completely personalized design.
- It is possible to consider mixed directional behaviors, so that one area of the soundboard is printed with one direction and another part with another direction.
- Although the SolidWorks design software is not able to generate the particularities of the printing directions and other parameters of additive manufacturing, it does allow for establishing an initial approximation of the vibrational behavior of the harmonic cap, in case one desires to modify parameters, such as the wandering.
- The specimen printed at 0° generated outliers, causing vibration amplitude at some points quite far from the average of the rest of the specimens.
- The largest displacements in the minor lobe are achieved with a printing direction of 90°, since it transmits the vibration from the major lobe to the minor lobe, as occurs with the traditional wooden caps.
- The displacements at the second point, below the top plate, are enhanced with an impression direction perpendicular to the direction of the strings, i.e., 0°. Concretely, this top concentrates the vibration on the major lobe, approaching the waist of the top.
- At frequencies of 120–300 Hz, an impression angle of −45° causes greater average displacements in the left zone, where the thickest strings would be found and with which bass sounds are generated, so if in addition to that direction, the reinforcement in the central zone of the major lobe is increased.
- It is important to continue the research in this field so as to define quality indexes at a later stage, and to carry out a study according to these parameters.

In general terms, with the data obtained, it can be concluded that additive manufacturing technology with low-cost machines can be used for the design and manufacture of an acoustic guitar. This allows for carrying out different designs, manufacturing test tops quickly, reducing generation and evaluation times. On the other hand, it is considered of interest to develop a comparative analysis with a conventionally manufactured wooden top.

## Figures and Tables

**Table 1:** Printing directions (angles).

*Angles*				
0°	90°	+45°	−45°	±45°
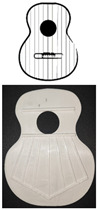	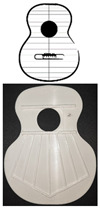	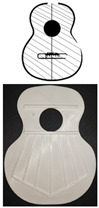	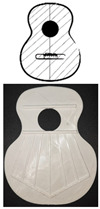	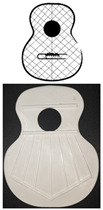

**Table 2:** Vibrometer data from Wintact WT63B.

Acceleration	0.1~199.9 m/s^2^
Velocity	0.1~199.9 mm/s
Displacement	0.001~1.999 mm
Accuracy vibration displacement	0.01~0.02 mm, ≤±10%, ≥0.02 mm, ≤±5%
Accuracy vibration speed	0~2.0 mm/s, ≤±10%, ≥2.0 mm/s, ≤±5%
Accuracy vibration acceleration	0~2.0 mm/s^2^, ≤±10%, ≥2.0 mm/s^2^, ≤±5%
High frequency	1 KHz~15 KHz (HI)
Low frequency	20 Hz~1 KHz (LO)

**Table 3:** Standard deviation. Displacement (mm).

	Point				
Mode	1	2	3	4	5
1	0.016	0.051	0.019	0.052	0.043
2	0.011	0.031	0.009	0.014	0.023
3	0.021	0.314	0.017	0.005	0.017
4	0.027	0.140	0.013	0.012	0.025
5	0.010	0.012	0.014	0.015	0.002

**Table 4:** ANOVA Table.

Point 1 the Whole Sample					
Source	SS	df	MS	F	Prob > F
**Columns**	0.04762	4	0.01191	34.61	9.99967 × 10^−09^
**Error**	0.00688	20	0.00034		
**Total**	0.0545	24			
**Point 2**					
**Columns**	0.052	4	0.013	0.53	0.7137
**Error**	0.48864	20	0.02443		
**Total**	0.54063	24			
**Point 3**					
**Columns**	0.03654	4	0.00914	39.39	3.24871 × 10^−09^
**Error**	0.00464	20	0.00023		
**Total**	0.04118	24			
**Point 4**					
**Columns**	0.02059	4	0.00515	7.6	0.0007
**Error**	0.01354	20	0.00068		
**Total**	0.03412	24			
**Point 5**					
**Columns**	0.04328	4	0.01082	16.02	5.00595 × 10^−06^
**Error**	0.01351	20	0.00068		
**Total**	0.05679	24			

**Table 5:** ANOVA table.

Point 1 No 0° Sample					
Source	SS	df	MS	F	Prob > F
**Columns**	0.03393	4	0.00848	47.12	2.55311 × 10^−08^
**Error**	0.0027	15	0.00018		
**Total**	0.03663	19			
**Point 2**					
**Columns**	0.0344	4	0.0086	6.25	0.0036
**Error**	0.02063	15	0.00138		
**Total**	0.05502	19			
**Point 3**					
**Columns**	0.02915	4	0.00729	32.07	3.44303 × 10^−07^
**Error**	0.00341	15	0.00023		
**Total**	0.03256	19			
**Point 4**					
**Columns**	0.01214	4	0.00304	4.97	0.0094
**Error**	0.00917	15	0.00061		
**Total**	0.02131	19			
**Point 5**					
**Columns**	0.03225	4	0.00806	15.52	3.28682 × 10^−05^
**Error**	0.0078	15	0.00052		
**Total**	0.04005	19			

**Table 6:** Relative error FEM vs. test.

			Modes									
Point	PD		M2 vs. M4	M5	M2 vs. M4	M5	M2 vs. M4	M5	M2 vs. M4	M5	M2 vs. M4	M5
1	0°	FEM	0.159	0.082	0.159	0.082	0.159	0.082	0.159	0.082	0.159	0.082
		ED	0.1	0.074	0.166	0.102	0.157	0.077	0.120	0.087	0.138	0.086
		RE	−59.6	−12.0	4.2	18.7	−1.3	−6.9	−32.4	5.3	−15.6	3.6
2	90°	FEM	0.017	0.070	0.017	0.070	0.017	0.070	0.017	0.070	0.017	0.070
		ED	0.381	0.055	0.159	0.046	0.044	0.079	0.062	0.063	0.071	0.071
		RE	95.4	−27.6	88.9	−52.3	60.2	10.9	71.5	−11.5	75.1	1.0
3	+45°	FEM	0.021	0.070	0.021	0.070	0.021	0.070	0.021	0.070	0.021	0.070
		ED	0.083	0.05	0.074	0.08	0.052	0.086	0.056	0.064	0.071	0.069
		RE	74.1	−40.0	70.8	12.5	58.4	18.6	61.4	−9.4	69.5	−1.5
4	−45°	FEM	0.113	0.066	0.113	0.066	0.113	0.066	0.113	0.066	0.113	0.066
		ED	0.140	0.095	0.128	0.064	0.128	0.093	0.132	0.083	0.106	0.066
		RE	18.9	30.0	11.0	−3.9	11.0	28.8	13.7	19.9	−7.4	−0.8
5	±45°	FEM	0.017	0.070	0.017	0.070	0.017	0.070	0.017	0.070	0.017	0.070
		ED	0.112	0.078	0.056	0.073	0.070	0.075	0.045	0.072	0.068	0.078
		RE	84.2	9.2	68.6	3.0	74.9	6.2	60.7	1.7	74.1	9.2

FEM = FEM displacement (mm); ED = experimental displacement (mm); RE = relative error (%); PD = print direction.

## Data Availability

The original contributions presented in the study are included in the article, further inquiries can be directed to the corresponding author.
